# Multiplexed single-tier immunoassay for improved Lyme disease diagnosis across all disease stages

**DOI:** 10.21203/rs.3.rs-8502997/v1

**Published:** 2026-02-09

**Authors:** Phillip Stafford, Holly Ahern, John Aucott, Alison Rebman, Elizabeth J. Horn, Marianne Hathaway, Madeleine Saunders, Nicole R. Hasenkampf, Monica E. Embers

**Affiliations:** 1 Arizona State University, School of Life Sciences, Tempe, AZ; 2 State University of New York, Adirondack, Queensbury, NY; 3 Johns Hopkins University School of Medicine, Baltimore, MD; 4 The Lyme Disease Biobank, Portland, OR; 5 Tulane National Biomedical Research Center, Covington, LA

## Abstract

Lyme disease is the most common vector-borne infection in North America and Europe, yet current two-tier serologic testing shows poor sensitivity during early infection when antibiotic treatment is most effective. We developed and validated a single-tier multiplex immunoassay that combines ten *Borrelia burgdorferi* antigens with machine-learning classification to detect antibody responses across all disease stages. A total of 364 cases were obtained, which included well characterized prospective blood samples obtained during early infection. In a cohort of samples from the Johns Hopkins University SLICE studies, the classifier identified all 30 early Lyme disease cases and their 1–3-month post-treatment follow-up samples. To assess generalizability, an independent EU laboratory synthesized the antigens *de novo* and a distinct 259-person cohort was evaluated on newly built assays. The classifier achieved an AUC of 0.98 in both cohorts. This single-test platform delivers quantitative serologic classification using standard clinical laboratory instrumentation, addressing critical gaps in Lyme disease diagnosis.

## Introduction

*Borrelia burgdorferi* sensu lato spirochetes, transmitted by *Ixodes* ticks, cause Lyme disease^1^, the most common vector-borne infection in North America and Europe with an estimated 476,000 annual U.S. cases^2^ and over 200,000 cases in Western Europe^3^. Following a tick bite, the bacteria can disseminate to joints, nerves, and heart, triggering inflammatory responses that produce neurologic, cardiac, and arthritic complications^4^.

Early diagnosis depends primarily on provider recognition of an erythema migrans (EM) rash^5^, yet this characteristic rash is absent or atypical in many patients^6^. Most present with nonspecific symptoms indistinguishable from other febrile conditions, delaying treatment and increasing risk of disseminated infection. With 15–20% of ideally diagnosed and treated patients experiencing symptoms lasting beyond six months, early accurate detection is critical^7,13^.

Current two-tier serologic testing detects only 28–50% of early-stage cases during the first 3–4 weeks when antibiotic treatment is most effective^7–9^. This poor sensitivity in early infection, precisely when intervention has the greatest impact, represents a major gap in clinical practice.

We hypothesized that multiplexing diverse *Borrelia* antigens with machine learning classification could capture the evolving immune signature across disease stages. Here we report development and validation of a single-tier immunoassay combining ten *B. burgdorferi* antigens with a tree-based classifier, which achieves high sensitivity and specificity from symptom onset through late disease, with analytical reproducibility across independent laboratories.

## Results

### Cohort characteristics and study design

**Figure 1:**
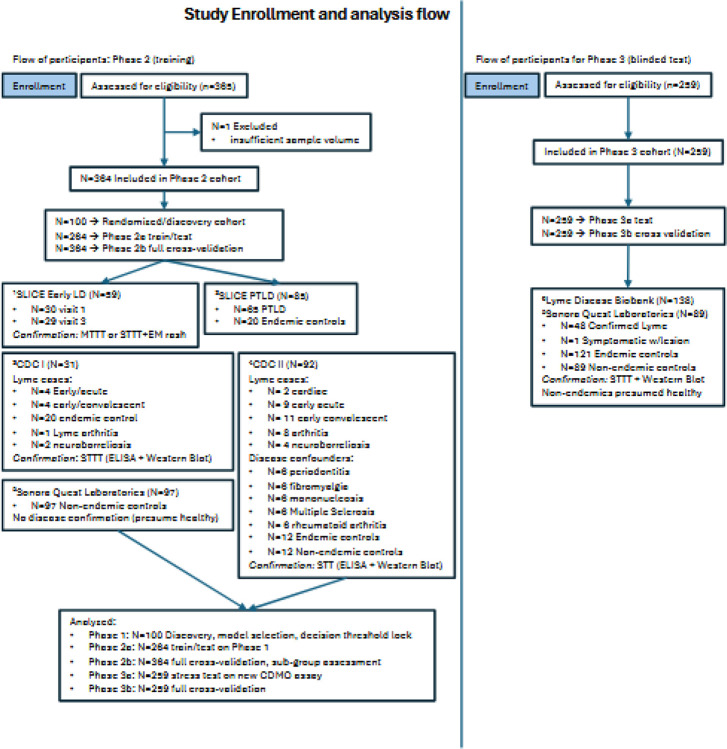
CONSORT diagram for sample collection and allocation. The training cohort (N=364) comprised samples from the Center for Disease Control (CDC) reference collections (called CDC I and II), Johns Hopkins Slice studies (SLICE I and III), and Sonora Quest Laboratories (SQL). Samples were randomized into Phase 1 discovery (N=100), Phase 2a holdout (N=264), and Phase 2b being the full internal validation (N=364). The independent test cohort (N=259) was used for the blinded Phase 3a validation and Phase 3b cross-validation. One sample from SLICE I paired visits was excluded due to insufficient volume. Superscripts denotate sample sources ^1^SLICE I study^14,2^SLICE III study^12,3^CDC I,^4^CDC II^10,5^Sonora Quest Laboratories, Tempe, AZ,^6^Bay Area Lyme Foundation, Portola Valley, CA.

To minimize bias and overtraining, we implemented a multi-phase discovery and validation design with pre-specified decision points (CONSORT diagram, Figure 1). We assembled 364 geographically diverse patient samples: two CDC reference collections^10,11^ (CDC I: 11 cases, 20 controls; CDC II: 32 cases, 6 each of 6 different disease confounders, 12 endemic and 12 non-endemic controls); 97 non-endemic controls from Sonora Quest Laboratories (Phoenix, AZ); 145 early LD and PTLD samples from the Johns Hopkins University “Study of Lyme disease Immunology and Clinical Events” (SLICE).^12^ This sample set consisted of 30 patients with early Lyme disease (early LD) and physician-documented erythema migrans rash with paired 1–3 month follow-up, 65 post treatment Lyme disease (PTLD) cases^17^, and 20 endemic controls without a clinical or serologic history of Lyme disease.

Serum samples were processed by Luminex^®^ assay, capturing IgG and IgM for each of the 10 antigens. The ratio between case and control shows the mean reactivity per antigen is generally higher in case than control. However, C6-IgG alone recapitulated published ELISA performance^13^, and performed well for some subgroups but demonstrated low sensitivity for others. This result supported the need for multiple antigens to capture diverse anti-*Borrelia* response

### Phase 1: Discovery

In Phase 1 (discovery), we randomly allocated 50 cases and 50 controls to train and rank 15 classification algorithms, selecting one model and locking both the classifier and decision threshold using only Phase 1 data. The full evaluation of the discovery cohort for all 15 classifier models is shown in Supplemental Table 1.

### Phase 2a: Holdout validation

After locking the XGBoost model, the decision threshold (0.377), and confirming use of all 20 features using the Phase 1 data (N=100), we then tested these 100 cases using the remaining 264 holdout samples as training. This strict separation ensures the model never encountered these samples during development, obtaining accuracy of 0.98 (CI 0.93–1.00).

### Phase 2b: Internal validation across disease stages and confounders

We evaluated the locked classifier across 364 samples, encompassing 197 two-tier-confirmed Lyme disease cases (all clinical stages), and 167 controls (108 healthy individuals, 59 with febrile illnesses or Lyme-mimicking conditions). To assess overfitting risk, we validated performance using three cross-validation strategies (5-fold, 10-fold, and leave-one-out) at the locked 0.377 threshold. Supplemental Table 1 shows results from all 15 candidate classifiers to address model selection bias.

The Johns Hopkins SLICE early LD samples^14^ collected at time of diagnosis and before initiation of treatment, (visit 1) and 1–3 months post-completion of antibiotic treatment (visit 3) are a unique aspect of this study. The assay identified 30/30 visit 1 patients and 29/29 visit 3 timepoints compared to 11/30 and 22/29 using STTT (EIA with confirmation by Western blot, performed by Quest Labs) at the respective timepoints.

The SLICE PTLD cohort including 65 cases meeting the operational definition of post-treatment Lyme disease syndrome^14^ were tested The assay achieved 95% sensitivity (92–100%) and 97% specificity (92–100%) vs. 43% sensitivity achieved by STTT^15^. Depending on time of diagnosis and first antibiotic treatment, 15 – 30%^16 17^ of Lyme disease patients may develop prolonged debilitating symptoms; therefore, this performance enables objective assessment of patients with continuing manifestations. Because the JHU SLICE clinical studies have had a profound impact on Lyme disease clinical research, we highlighted the analytical performance in Table 1. Heatmaps showing signal intensity for patients’ antibodies against each antigens is provided in Supplemental Figure 1.

Overall Phase 2b performance achieved a sensitivity of 0.93 (0.89–0.95), specificity of 0.91 (0.87–0.95), positive likelihood ratio 10.3, and negative likelihood ratio 0.08 (Table 2).

**Figure 2:**
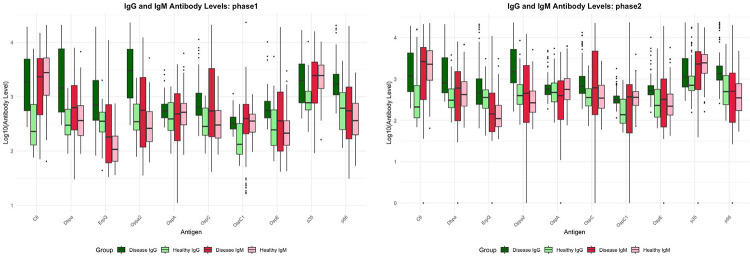
The difference in signal between healthy and disease for the given cohort for Phase 1 (N=100) and Phase 2 (N=364) Luminex data. Dark and light green represent the IgG signals as detected by the 565nm fluorophore (phycoerythrin) on the mouse anti-human IgG (H+L) secondary; dark and light red represent the IgM signals, detected by secondary Goat Anti-Human IgM-PE. Each chart highlights

SHapley Additive exPlanations (SHAP) feature importance analysis from Phase 2b integrated data from samples spanning early infection through post-treatment disease. Features with highest predictive weight matched known immunodominant antigens at corresponding disease stages (Table 3), validating that the classifier captures genuine immune signatures rather than spurious correlations. The differences in reactivity between healthy and disease patient samples for each antigen is depicted in Figure 2, and the probability scores from the 15 different classifiers (Phase 2 and 3) is shown in Supplemental Figure 2.

### Phase 3a and 3b: Independent laboratory validation under manufacturing conditions

Phase 3a evaluated assay reproducibility during technology transfer from academia to a manufacturing environment. A European Contract Development and Manufacturing Organization (CDMO) was used to evaluate the diagnostic for potential manufacture. The company received only the DNA sequences for the antigens. They subsequently had the expression vectors generated and transfected into the *E. coli* host, then produced and purified the proteins. Peptides were synthesized and all antigens were conjugated to Luminex^®^ beads, followed by establishment of assay protocols, recruitment of 259 distinct case/control samples, performance testing on their own instrumentation, and returned of raw fluorescence data with anonymized identifiers. The CDMO had no access to training data, the classifier, or predictions.

A critical distinction must be made: Phase 3a represents a transferability stress test, not final product performance. This academic-to-manufacturing transition deliberately introduced multiple uncontrolled variance sources: different reagent lots, instruments, operators, peptide synthesis batches, protein expression systems, and sample populations. The locked decision threshold (0.377), optimized on the Phase 2b training samples, was intentionally not recalibrated to isolate the impact of manufacturing transfer.

To manage this uncalibrated technical variance while prioritizing clinical safety, we implemented a conservative three-zone strategy: positive (>0.377), equivocal (0.20–0.377), and negative (<0.20), targeting a negative predictive value (NPV) ≥0.95. Under these deliberately uncalibrated conditions, Phase 3a achieved an AUC of 0.93 (95% CI: 0.88–0.99), sensitivity 0.89 (0.77–0.95), and specificity 0.94 (0.90–0.96). This represents worst-case performance with the variance from the new manufacturing methods impacting the efficacy of the training data.

Phase 3b represents a true external validation: after unblinding, we applied the locked classifier directly to case and control samples obtained from the Lyme Disease Biobank (Bay Area Lyme Foundation) and Sonora Quest Laboratories using the standard 0.377 threshold with no equivocal zone. Phase 3b achieved AUC 0.98 (0.96–1.00), sensitivity 0.90 (0.78–0.97), specificity 0.98 (0.95–0.99), positive likelihood ratio 45, and negative likelihood ratio 0.10, generally matching the Phase 2b internal validation, confirming the assay maintains diagnostic accuracy when rebuilt from antigen information only.

### Antigen selection and assay development

We selected ten antigens to capture *B. burgdorferi’s* dynamic expression patterns and temporal evolution of host antibody responses^18,22,23^. Antigen expression characteristics are shown in Table 1. Early infection antigens OspC and DbpA are expressed during tick transmission and initial colonization, eliciting detectable IgM and IgG responses within days of symptom onset^24–26^. OspA, though primarily expressed in the tick midgut, generates antibodies in patients with prolonged infection or antibiotic-refractory disease^27,28^. VlsE and its conserved C6 peptide detect antibodies against this variable surface lipoprotein throughout infection, providing pan-stage reactivity^13,29^.

*B. burgdorferi* evades immune surveillance by downregulating OspC post-dissemination while upregulating alternative surface proteins, creating stage-specific antigenic profiles^28^. We therefore included antigens expressed during tissue colonization and persistence: OppA2 (oligopeptide permease A2), P35^30,31^, DbpA (decorin-binding proteins mediating tissue adhesion)^26^, and P66 (outer membrane porin generating sustained antibody responses)^32^.

This design addresses the fundamental challenge in Lyme serology: individual patients mount highly variable responses depending on bacterial load, tissue tropism, infection duration, and host immune genetics^33^. The machine learning classifier integrates reactivity across this antigen array to reconstruct patient-specific immune signatures, creating a composite biomarker that captures the heterogeneous host-pathogen interaction underlying Lyme disease.

### Model selection and decision threshold lock

We evaluated 15 classification algorithms on Phase 1 data using 5-fold, 10-fold, and leave-one-out cross-validation (Supplemental Table 1). Tree-based ensemble methods consistently outperformed linear, non-linear, statistical, rules-based and other binary classification models. We selected XGBoost and locked all hyperparameters (120 estimators, learning rate 0.015, max depth 3, gamma 1.5, min child weight 5, subsample 0.9) and the decision threshold (0.377, optimized by Youden’s index^34^) using only Phase 1 data (Supplemental Figure 3).

### Feature Selection and Ranking

We applied Recursive Feature Elimination (RFE) with leave-one-out cross-validation to identify essential antigens. There are 20 features: 10 antigens and two isotypes. The 20-feature panel consistently outperformed smaller subsets across validation cohorts, confirming that every feature contributes non-redundant diagnostic information (Supplemental Figure 4).

We wished to rank each feature by importance to correlate known immune response with our observations. We employed three independent ranking methods: ensemble voting across five different classifiers (SVM, XGBoost, C5.0, gbm, SVM), XGBoost-only^35^, and SHAP^36^ values.

The classifier method tries to optimize for AUC, whereas the SHAP method quantifies marginal contribution to individual predictions per feature (Supplemental Table 2, columns 14–16). In early disease (v1), OspC1-IgG and OppA2-IgG carried high predictive weights, consistent with immunodominance during early dissemination and late infection, respectively. DbpA-IgM and C6-IgM showed minimal contributions, reflecting their transient early kinetics. Feature importance matching known *Borrelia* immunology confirms the model captures genuine immune signatures rather than spurious correlations. Ranking variations between the different methods reflect algorithmic differences rather than biological disagreement: tree-based classifiers assess information gain from sequential splits, while SHAP provides model-agnostic measurement of feature contribution across all possible feature combinations^34–36^.

Supplemental Table 2 reports comprehensive single-antigen performance metrics including AUC, sensitivity, specificity, Youden’s index, case-control signal distributions (mean fluorescence intensity, effect size), statistical significance (t-tests, Cohen’s d), and feature importance rankings from all three methods.

### Probability score characteristics and calibration

Classifier scores demonstrated bimodal distributions: correctly classified samples concentrated at high confidence (near 0 or 1), while misclassifications clustered near the decision boundary (Supplemental Figure 5). Confidence scores differed significantly between correct and incorrect classifications in Phase 2b (FP vs. TP: p=8.4×10^−9^; FN vs. TN: p=1.92×10^−16^) and Phase 3b (p=2.26×10^−25^ and p=1.80×10^−34^ respectively), indicating that most correct predictions carry high certainty and incorrect predictions carry lower certainty. However, larger studies are needed to define optimal confidence thresholds.

Effect size analysis of continuous probability scores revealed exceptional group separation (Supplemental Table 2). Cohen’s d value ranged from 2.73 to 6.02 across validation phases, indicating large effect sizes substantially exceeding conventional thresholds (0.8). Phase 3b achieved the strongest discrimination (disease mean 0.869 vs. control mean 0.022, Cohen’s d=6.02, Glass’s Δ=10.46), confirming robust separation even when using control variance alone.

Calibration metrics demonstrated well-calibrated probability estimates: Brier scores <0.10 across all cohorts (substantially better than 0.25 threshold for clinically useful prediction), Brier Skill Scores approaching 1.0 (perfect calibration), and low expected calibration error using 10-observation bins. These metrics confirm the classifier produces not only accurate binary classifications, but probability estimates that accurately reflect true disease likelihood—a critical requirement for clinical decision-making^34^.

### Calibration performance and prediction confidence

Brier scores remained low across all validation phases (0.023–0.086), indicating well-calibrated probability estimates that accurately reflect true disease likelihood. Expected Calibration Error values were minimal (0.001–0.005), confirming that predicted probabilities closely matched observed outcome frequencies. Calibration curves (Supplemental Figure 6) demonstrate consistently high-quality probability estimates across the full score range.

The proportion of predictions near the decision boundary (indeterminate region around 0.377) decreased progressively: 7% in Phase 1 discovery, 3.4% in Phase 2b, and 1.2% in Phase 3b. This reduction reflects increasing model confidence with larger validation cohorts, with 98.8% of Phase 3b predictions providing unambiguous classification. Nevertheless, formal assessment of prediction confidence intervals in prospective studies could further aid clinical interpretation. By mapping model probabilities to antigen-level SHAP contributions (Supplemental Figure 7), clinicians can interpret each positive result as a quantitative immunologic signature rather than a binary outcome, an approach that may enhance transparency and regulatory acceptance of machine learning diagnostics.

### Clinical decision curve analysis

**Figure 3:**
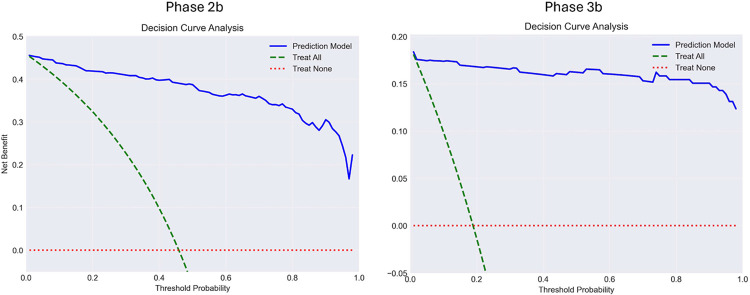
Decision curve analyses. Net benefit approaches to the evaluation of prediction models, molecular markers, and diagnostic tests. Decision curve showing expected net benefit per patient as a function of the treatment threshold probability. The model-based curve incorporates the predicted probability of Lyme disease, while “Treat all” and “Treat none” represent the two extreme strategies. Threshold probabilities correspond to different assumptions about the cost and side effects of doxycycline relative to the benefit of correctly treating Lyme disease. Net benefit values are expressed as true-positive equivalents (top axis) or monetary/utility units (right axis) for direct interpretation. Phase 2b data are shown on left, Phase 3b data shown on right.

We evaluated clinical utility using decision curve analysis, which quantifies net benefit across varying treatment threshold probabilities (Figure 3). Net benefit is calculated as: [sensitivity × prevalence] − [(1−specificity) × (1−prevalence) × (p_t/(1−p_t))], where p_t represents the threshold probability at which a clinician would initiate treatment^37^.

In Phase 2b, the diagnostic model provided substantial net benefit compared to treat-all or treat-none strategies across threshold probabilities from 0.15 to 0.85, with maximum benefit at 0.30–0.35, closely matching the locked decision threshold of 0.377. In Phase 3b, the model maintained superior net benefit across an even broader range (0.05–0.95), with maximum benefit at 0.50–0.55, reflecting this cohort’s improved sensitivity and specificity. The model demonstrates clinical value across nearly all reasonable treatment thresholds, indicating robust utility across diverse clinical settings and physician risk preferences.

## Discussion

This multiplexed immunoassay captures stage-specific immune heterogeneity through biologically interpretable features and locked decision thresholds. The single-tier format achieved 100% sensitivity in early Lyme disease patients – a critical window when antibiotic treatment is most effective. Since treatment efficacy declines substantially after bacterial dissemination, detection at hyperacute presentation could prevent progression to chronic debilitating symptoms^38–41^.

### Comparison to existing diagnostics

Recent single-tier approaches, including the Hybrid Lyme ELISA, demonstrated 90–94% sensitivity for patients with unequivocal erythema migrans^42^. Steere *et al*. and others report that the EM rash appears in 70% or less of patients^6,43,44^; however, only the classic bulls-eye appearing rash is widely recognized as a clinical sign for Lyme disease. A majority of patients present with an atypical rash or no rash^44^, which adds to diagnostic uncertainty. Our cohort addresses this gap by including patients without EM rash, patients presenting with only systemic symptoms, and individuals across the disease spectrum from early acute to chronic infection.

For PTLD cases from the Johns Hopkins SLICE study, the assay achieved 95% (94–100%) sensitivity, compared to 43% for standard two-tier testing among a similar set of patients from this same cohort^15^. While PTLD lacks curative therapy, improved diagnostic precision validates patient symptoms, supports standardized research case definitions, and facilitates mechanistic studies^39,41,45^.

The CDC provides a panel of samples, collected from early and late disease patients, from confounders, and from endemic controls. The CDC published the initial assessment of diagnostic testing of their own samples, confirming STTT results have high specificity but 30–40% sensitivity for early infection^10,11^. This panel of samples was used by our laboratory multiple times, for early development. Although antibodies are stable when frozen, repeated freeze-thaw cycles may lower the amount of intact antibody. Results from the CDC cohorts show lower repeatability than other cohorts (technical replicate CV>80% average vs. <20% for samples with <3 freeze-thaw cycles). It is possible that degradation of this sample set could account for lower signals from the CDC samples. These are a limited resource and repeat batches are difficult to obtain.

### Clinical impact in endemic and non-endemic settings

The assay’s performance characteristics translate to meaningful clinical benefit. Untreated *B. burgdorferi* infection disseminates within 1–2 weeks, affecting approximately 50% of cases and underscoring the importance of early accurate diagnosis. In non-endemic regions, the assay’s high negative predictive value (0.95, 95% CI: 0.91–0.97) could substantially reduce unnecessary antibiotic prescriptions while ensuring treatment for true cases. The PPV ranges from 0.35 where Lyme prevalence is 5% to PPV of 0.10 when Lyme prevalence is 1%.

### Diagnostic utility in post-treatment Lyme disease

The clinical utility of PTLD diagnosis differs fundamentally from early disease detection. Four NIH-sponsored randomized controlled trials evaluating extended antibiotic therapy for PTLD showed limited sustained benefit or adverse events. While the STOP-LD trial demonstrated transient improvement in fatigue scores^27^, benefits were not sustained. Current CDC, NIAID, and IDSA guidelines recommend against prolonged antibiotic therapy for PTLD, emphasizing instead symptomatic management and supportive care^46–48^.

The value of improved PTLD diagnosis therefore lies in: (1) validation of patient symptoms and reduction of diagnostic uncertainty, (2) standardized case definitions for research cohorts, (3) facilitation of mechanistic studies investigating pathophysiology, and (4) exclusion of alternative diagnoses requiring different management when effective treatments exist. While PTLD remains clinically heterogeneous, our results demonstrate that immune signatures in these patients are reproducibly distinct from healthy controls across independent cohorts. The assay’s utility is not to confirm persistent infection requiring antibiotics, but to provide objective serologic classification that supports standardized research criteria and reduces diagnostic ambiguity in both clinical and research settings.

### Biological basis of classifier performance

The assay’s performance reflects fundamental *B. burgdorferi* biology. Unlike pathogens presenting consistent antigenic targets, *Borrelia* undergoes antigenic variation and stage-specific gene regulation, creating temporal immune heterogeneity that single-antigen tests cannot capture. SHAP feature importance analysis revealed biologically coherent patterns: OspC-IgM and DbpA-IgM dominated in early disease, consistent with rapid antibody kinetics during initial dissemination; C6-IgG, VlsE-IgG, and OppA2-IgG dominated in disseminated disease, reflecting persistent expression during tissue colonization; and persistent IgG responses to multiple antigens characterized PTLD cases.

This adaptive antigen weighting enables the classifier to accommodate individual immune response heterogeneity that may be driven by bacterial strain differences, MHC haplotype variation, infection route, and treatment timing. This flexibility, possible only with multiplexed antigens, ensures performance across heterogeneous populations and infection stages.

The high specificity (90–96%) despite potential cross-reactivity from other spirochetal infections and autoimmune conditions demonstrates successful pattern recognition of Lyme-specific immune signatures. The classifier distinguished syphilis, a spirochetal infection caused by *Treponema pallidum*, and correctly classified patients with rheumatoid arthritis and systemic lupus erythematosus as controls.

### Clinical implications and interpretability

The probabilistic output provides quantitative information beyond binary classification. Scores near the decision threshold may indicate very early infection before full seroconversion, waning responses in treated patients, weak responses in immunocompromised individuals, or cross-reactive responses requiring clinical correlation. This biological interpretability (i.e. the classifier’s alignment with known immunologic principles) provides confidence that the model captures genuine disease biology rather than spurious correlations.

Decision curve analysis demonstrated substantial net benefit across clinically relevant threshold probabilities in both Phase 2b (0.15–0.85) and Phase 3b (0.05–0.95), indicating robust clinical utility across diverse settings and physician risk preferences. In non-endemic regions, the high negative predictive value (0.95, 95% CI: 0.91–0.97) could reduce unnecessary antibiotic prescriptions while ensuring treatment for true cases. Table 2 demonstrates maintained clinical utility across prevalence ranges from endemic to non-endemic regions.

### Technological innovation and broader implications

The critical advance lies in strategic integration of rationally selected antigens with machine learning to capture immune heterogeneity across disease stages in a clinically deployable format. By establishing locked decision thresholds during validation, the approach prevented overfitting while maintaining biological interpretability. This addresses the fundamental limitation that single thresholds applied to individual antigens cannot capture temporal and individual heterogeneity of immune responses.

This methodology may extend to other vector-borne diseases with inadequate diagnostic sensitivity or specificity. Diagnosis of babesiosis^49^, ehrlichiosis, anaplasmosis^50^, and emerging tick-borne diseases^51^ may benefit from multiplexed antigen panels with machine learning optimization.

### Regulatory pathway

We are engaging with the FDA to pursue regulatory approval for commercial manufacturing and clinical availability.

### Limitations

This retrospective study utilized established biobanks and research cohorts. While enabling comprehensive validation across disease stages and geographic regions, prospective validation in consecutive clinical enrollments under routine diagnostic conditions is essential and is currently underway. Phase 3a demonstrated manufacturing reproducibility but was not designed as a blinded clinical trial with predetermined performance criteria; future prospective studies with prespecified thresholds and clinical adjudication of discordant results are necessary for regulatory evaluation.

Our cohort included limited representation of immunocompromised patients, pediatric populations, and individuals with tick-borne co-infections who may exhibit altered immune responses. The spectrum of autoimmune and inflammatory conditions was not exhaustively represented. Validation in these populations is warranted before generalization to all clinical contexts.

The assay demonstrated sensitivity in both early Lyme and PTLD patients, indicating persistent antibody responses in most individuals with prolonged post-treatment symptoms. However, positive serology does not distinguish active infection from resolved infection with persistent antibodies, limiting utility for treatment response monitoring. Complementary approaches such as direct pathogen detection would be needed to address this clinical question.

Our cohort encompasses North American *B. burgdorferi sensu stricto*. Other Borrelia genospecies across the *B. burgdorferi sensu lato* complex are known to cause Lyme borreliosis in Europe and Asia. Geographic strain diversity and antigenic polymorphism may affect test performance. However, current tests do not differentiate genospecies and it is possible that the cohorts tested during development included cases caused by other *Borrelia* genospecies (*B. mayonii, B. miyamatoi*, *B. garinii*)^52^. Validation with European (*B. afzelii*, *B. garinii*) and Asian (*B. bavariensis*) species remains necessary to confirm performance across the *B. burgdorferi* sensu lato complex.

While training (N=364) and independent test (N=259) cohorts were adequate for development and initial validation, larger multicenter prospective studies are needed to establish performance in low-prevalence populations, define performance in specific subgroups, and assess integration into clinical workflows with varying pre-test probabilities. These are being obtained now through contractors and specimen brokers, as well as the ongoing prospective studies.

The decision threshold (0.377) was optimized using Youden’s index, balancing sensitivity and specificity without accounting for varying clinical costs of false positives versus false negatives. Context-specific threshold optimization based on clinical utility may be warranted for different use cases, eg. lower cutoffs to maximize sensitivity in high-prevalence settings and higher cutoffs for screening in low-prevalence populations.

This assay detects antibody responses rather than direct evidence of active infection, and cannot distinguish recent from past infection absent clinical context. Some reports suggest that low levels of antibodies correlate with worse outcomes, suggesting that high sensitivity is required to find patients most in need of immediate treatment^53^. Integration with clinical presentation (noting that many if not most patients may present with no or atypical EM rash^6,43 44^), epidemiologic risk factors, and temporal symptom progression is essential for appropriate interpretation. As with all serologic tests, diagnostic specificity depends on training data quality. The classifier identifies immune patterns characteristic of laboratory-confirmed cases distinguished from cross-reactive patterns in diverse controls.

Despite these limitations, the rigorous validation approach—including independent cross-laboratory testing and locked model methodology—provides a strong foundation for prospective clinical validation and regulatory evaluation.

## Conclusion

We report the development and validation of a single-tier serologic diagnostic for Lyme disease that addresses key unmet clinical needs^9,54^. Although enrolling patients at the time of diagnosis and antibiotic initiation is challenging, evaluation of this early-acute cohort is essential for developing an assay capable of detecting infection at the most treatable stage. The diagnostic demonstrated high sensitivity at the earliest time point, with similarly robust performance at subsequent visits, defining a window of positivity that enables timely and effective antibiotic intervention. Notably, the assay achieved 95% sensitivity in post-treatment Lyme disease compared with 43% for STTT. By integrating ten biologically selected antigens with a machine-learning classifier, the test delivers consistent performance across the full disease spectrum while eliminating the complexity, subjectivity, and delays associated with reflect two-tier algorithms.

Independent laboratory validation under manufacturing conditions demonstrated reproducibility using only antigen DNA sequences. This analysis integrated independent reagent lots, equipment, protein expression/purification/bead conjugation, and a new sample cohort. Results confirm the underlying biology translates reliably from an academic to a more commercial setting. The transparent, interpretable model architecture, with SHAP values mapping predictions to known immunologic principles, provides regulatory agencies and clinicians mechanistic confidence that classifications reflect genuine immune signatures rather than algorithmic artifacts.

The clinical impact is immediate: early detection enables antibiotic intervention before bacterial dissemination, preventing progression to chronic debilitating disease. For symptomatic post-treatment patients, objective serologic classification supports clinical management and research standardization. Beyond Lyme disease, this work establishes a generalizable framework of multiplexed antigens capturing temporal immune heterogeneity through biologically constrained machine learning. This framework may be applicable to other complex vector-borne infections where single biomarkers inadequately reflect host-pathogen dynamics. As Lyme disease incidence rises globally, this validated platform provides a pathway from research innovation to clinical implementation that balances diagnostic performance, analytical rigor, and regulatory requirements for widespread adoption.

## Methods

### Study design and participants

All study protocols adhered to the Declaration of Helsinki. Prior studies suggested broad Lyme antigen panels could perform well across early, convalescent, and late disease stages. This multicenter study was conducted under IRB-approved protocols at participating institutions with written informed consent from all participants.

IRB approvals: Tulane University: “A Multiplex Platform for Lyme Disease Diagnosis and Treatment Response”; Johns Hopkins University: NA_00011170, IRB00035457 (SLICE); Lyme Disease Biobank: Participants from East Hampton (EH) and Wisconsin (WI) were enrolled under Advarra IRB protocol Pro00012408 and Marshfield Clinic Research Institute IRB protocol SCH20216, respectively.

Inclusion criteria - Lyme disease cases (early/disseminated): Age ≥18 years, physician-documented erythema migrans rash ≥ 5cm at time of enrollment (SLICE), and/or systemic symptoms plus positive standard two-tier test and confirmed tick exposure (LDB).

Inclusion criteria - PTLD cases (SLICE)^17^: Age ≥18 years, physician-documented prior CDC-confirmed or probable Lyme disease (EM rash, neurologic Lyme disease, carditis, or late Lyme arthritis) based on medical record review, completed recommended antibiotic treatment for early or late Lyme disease, fatigue, musculoskeletal pain, or neurocognitive complaints persisting ≥6 months and beginning within 6 months of initial diagnosis (per IDSA definition).

Inclusion criteria - healthy controls: Age ≥18 years (≥10 years for LDB Phase 3 validation cohort), health status documentation. SLICE: negative two-tier serologic test and no self-reported history of Lyme disease diagnosis.

Exclusion criteria – early LD, PTLD and healthy controls: symptom duration >3 months (acute cases only), prior Lyme disease, prior treatment for current illness, autoimmune disorders, ME/CFS, fibromyalgia, major psychiatric conditions, Lyme vaccine history, sleep apnea, chronic liver disease, chronic neurologic disease, major psychiatric disease, recent cancer or malignancy, HIV or hepatitis, substance abuse.

Study scope: Limited to *Borrelia burgdorferi* sensu stricto naturally occurring in the United States.

### Antigen production and immunoassay development

Recombinant proteins (OspC, OspA, DbpA, OppA2, ErpQ, OspE, P35, P66) were expressed as glutathione-S-transferase (GST) fusion constructs in *E. coli* BL21 using pGEX 4T-1 vectors and purified by glutathione affinity chromatography. Synthetic peptides (OspC1, C6) were commercially synthesized. All antigens were coupled to Bio-Rad magnetic beads using the Bio-Plex Amine Coupling Kit.

The multiplexed immunoassay employed Luminex 200 technology with the following protocol: serum samples were diluted 1:200 and incubated with antigen-coupled beads for 1 hour, followed by detection with phycoerythrin-conjugated anti-human IgG and anti-human IgM secondary antibodies (Southern Biotech). Both IgG and IgM responses were measured for all ten antigens (20 features total). All samples were analyzed in duplicate with comprehensive quality controls.

### Study design and validation strategy

The analytical validation employed a three-phase design with prospectively locked algorithms to ensure genuine generalization rather than post-hoc optimization.

Phase 1: Discovery and algorithm selection (N=100). We randomly allocated 50 Lyme disease cases and 50 controls from the 364-participant training cohort to establish antigen performance benchmarks and select an optimal classification algorithm. We systematically evaluated 15 binary classification algorithms from functionally distinct families (details in Supplementary Materials) using 5-fold, 10-fold, and leave-one-out cross-validation. Features with correlation >0.90 were identified to assess covariance.

Following algorithm selection, we locked the XGBoost classifier with fixed hyperparameters and decision threshold (0.377, optimized by Youden’s index) before any analysis of holdout or independent validation datasets. No algorithm parameters, feature selection, or decision thresholds were modified based on subsequent phase performance—ensuring reported metrics represent true generalization.

Phase 2a: Holdout validation (N=264). The remaining 264 participants excluded from Phase 1 were used to test the locked model on the 100 Phase 1 holdout samples.

Phase 2b: Internal validation (N=364). We performed cross-validation across the complete 364-participant training cohort using 5-fold, 10-fold, and leave-one-out strategies to assess overfitting risk.

Phase 3a: Blinded technology transfer validation (N=259). An ISO-13485-certified European contract research organization independently synthesized antigens from DNA sequences, manufactured Luminex assays, procured 259 geographically distinct samples from the Lyme Disease Biobank, performed all testing, and returned only raw fluorescence data with anonymized identifiers. Phase 3a applied a conservative three-zone classification (positive >0.377, equivocal 0.20–0.377, negative <0.20) to manage uncalibrated technical variance from manufacturing transfer.

Phase 3b: Cross-validation of independent cohort (N=259). Following unblinding, we applied the locked US-trained classifier to European samples using the standard 0.377 threshold without equivocal zones.

### Performance metrics and calibration analysis

Standard diagnostic performance metrics included sensitivity, specificity, area under the receiver operating characteristic curve (AUC) with 95% confidence intervals (DeLong method), and likelihood ratios. Effect size for continuous probability scores was quantified using Cohen’s d (pooled variance) and Glass’s Δ (control variance only). Model calibration was assessed using Brier scores (values <0.25 indicate better-than-chance prediction; lower is better), Brier Skill Scores (comparing model to reference forecast; 1.0 is perfect), and Expected Calibration Error (ECE, quantifying alignment between predicted probabilities and observed frequencies using 10-observation bins). The percentage of predictions within ±0.1 of the decision threshold was calculated as an indeterminate zone metric. Decision curve analysis quantified net clinical benefit across varying treatment threshold probabilities.

## Supplementary Material

Supplementary Files

This is a list of supplementary files associated with this preprint. Click to download.

- SupplementalFigure6.pdf
- SupplementalInformationAutoRecovered.pdf

## Figures and Tables

**Table 1:** A subset of the Lyme disease cohort are of particular importance for a broadly high-performing multi-analyte diagnostic. The Johns Hopkins SLICE studies recruited clinically important cases. The prospective study followed patients from the earliest time of detection through weeks to months of post-treatment convalescence. The standard two-tier diagnostic test revealed a higher rate of false negatives (FN) for cases at early LD vs. convalescence. The current diagnostic was able to diagnose 30/30 early, antibiotic-naive Lyme cases and 29/29 of these patients 1–3 months post-treatment correctly. The same diagnostic was able to detect 63/65 of SLICE PTLD cases correctly.

Performance	Cross-validation N=364	SLICE I visit 1 N=30 case, 117 control	SLICE I visit 3 N=29 case, 117 control	SLICE III PTLD N=65 case, 117 control
AUC	0.98 (0.97–0.99)	1.00 (0.99–1.00)	1.00 (0.99–1.00)	0.9*9* (0.98–1.00)
sensitivity	0.93 (0.90–0.97)	1.00 (0.89–1.00)	1.00 (0.88–1.00)	0.95 (0.92–1.00)
specificity	0.91 (0.87–0.95)	0.97 (0.92, 0.99)	0.97 (0.92–0.99)	0.97 (0.92–0.99)
PPV	0.90 (0.85–0.94)	0.82 (0.73–0.95)	0.88 (0.73–0.95)	0.94 (0.86–0.98)
NPV	0.95 (0.91–0.97)	1.00 (0.97–1.00)	1.00 (0.97–1.00)	0.99 (0.95–1.00)
Accuracy	0.93 (0.89–0.95)	0.97 (0.93–0.98)	0.97 (0.93–0.99)	0.96 (0.94–0.99)

**Table 2:** overall Phase 2 (validation) performance. Left panels: case and control cohorts for Phase 2 (top) and Phase 3 (bottom) studies. “Truth” denotes the number of participants per subgroup, “Predict” indicates those correctly classified by the model. Right panels show confusion matrices and performance metrics, including AUC, accuracy, sensitivity, specificity, PPV, NPV, and the disease call rate. Phase 2a represents a 100-sample holdout set (50 cases, 50 controls) with the remaining 264 samples used for training. Phase 2b applies leave-one-out cross-validation (LOOCV) to the full 364-sample cohort. Phase 3a evaluates 259 blinded samples using the Tulane-trained 364-sample model against an independent CRO-generated dataset employing distinct antigens and instrumentation. Phase 3b applies LOOCV to the unblinded 259-sample cohort. On equivalent analyses, both Phase 2b and Phase 3b achieved an AUC of 0.98.

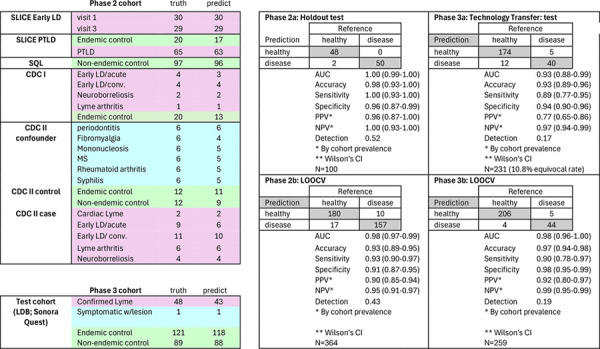

**Table 3:** Temporal and immunological characteristics of the ten-antigen Lyme diagnostic panel. Antigens were selected to capture immune responses across the complete disease spectrum, from initial dissemination through late persistent infection. OspC1 represents a conserved peptide region retaining cross-strain immunoreactivity, complementing full-length OspC which captures variable-region responses in early infection.

Antigen	Protein Class/Function	Stage of expression (Early -> Late)	Immune role/known reactivity	Ref
OspC1 peptide	Outer surface lipoprotein, tick-mammalian interface	Early (days 1–14)	Strong IgM/early IgG; hallmark of acute infection	
OspC full length	Outer surface protein C	Early (days 1–21)	IgM-to-IgG class switching marker	
OspA	Outer surface protein, tick midgut, re-expressed in joints	Late/antibiotic refractory	IgG; treatment failure/persistence marker	
OppA2	Oligopeptide permease, nutrient acquisition	Disseminated/late	Broad IgG reactivity	^18^
DbpA	Decorin-binding adhesin; tissue colonization	Disseminated/persistent	IgG; joint tissue tropism	^19^
ErpQ	Complement regulator-acquiring surface protein	Early disseminated	IgG; immune evasion marker	^20^
OspE	Complement factor H surface protein	Disseminated/persistent	Durable IgG response	
p35	Conserved immunogenic protein	Late/persistent	IgG; sustained titers	
p66	Outer membrane porin; adhesion	Late/persistent	IgG; minimal cross-reactivity	
C6 (VIsE) peptide	Invariant region of variable surface lipoprotein	Pan-stage	Broad IgG, decays post-treatment	^21^

## Data Availability

Raw data and machine learning code will be made available upon request following appropriate data sharing agreements to protect participant privacy. Code is available through GitHub: https://github.com/pstaffor/XGBoost-classifier.

## Data availability

The data that support the findings of this study are available at NCBI’s GEO data repository.
